# Sexual abuse and post-traumatic stress disorder in childhood adolescence and young adulthood

**DOI:** 10.12968/jfch.2025.2.3.112

**Published:** 2025-03-02

**Authors:** K Cleece, J Hill

**Affiliations:** 1 Social Work Research Lead, Lancashire and South Cumbria NHS Foundation Trust; 2 Senior Research Fellow, University of Central Lancashire

## Abstract

Child sexual abuse (CSA) is a pervasive global issue impacting children worldwide, with alarming statistics highlighting its prevalence. In England and Wales alone, a staggering 103,055 cases of CSA were recorded in the year ending 2022, marking a concerning 15% increase from figures reported in 2021. Studies indicate that approximately one in ten children under the age of 16 in the UK endure CSA, with girls experiencing it at a higher rate (1 in 6) than boys (1 in 20). The repercussions of CSA extend far beyond childhood, with victims more likely to grapple with mental health challenges in adulthood. Research suggests that about two-thirds of CSA victims experience mental or emotional issues as a result, underscoring the profound and lasting impact of such trauma. Specifically, there is a noted association between CSA and post-traumatic stress disorder (PTSD) and complex post-traumatic stress disorder (CPTSD), with repeated abuse heightening the likelihood of developing CPTSD. Boumpa et al. (2022) conducted a recent systematic review and meta-analysis aimed at further elucidating the correlation between CSA and lifetime psychiatric diagnoses, shedding light on this critical area of study. This commentary endeavours to conduct a critical appraisal of the methodologies employed in the review conducted by Boumpa et al. (2022) and to extend the discussion of the findings within the realm of social work practice.

## Introduction

Child sexual abuse (CSA) is a concern affecting children worldwide (WHO, 2022). In the year ending 2022 103,055 cases of CSA were recorded in England and Wales (Karsna & Bromley, 2023). Instances of CSA are increasing a 15% rise from the figures reported in 2021 (Karsna & Bromley, 2023). In the UK, an estimated one in ten children under the age of 16 experiences child sexual abuse, with statistics indicating this translates to 1 in 6 girls and 1 in 20 boys (Karsna & Kelly, 2018). Children who have experienced sexual abuse are more likely to present with mental health presentations in adulthood (Spataro, Mullen, Burgess, Wells, & Moss, 2004). Approximately two-thirds of victims of child sexual abuse are estimated to experience mental or emotional issues as a result (Brooker, Hughes, & Lucock, 2019). Specifically, there is a proposed correlation between post-traumatic stress disorder (PTSD) (Silva, Alpert, Munoz, Singh, Matzner, & Dummit, 2000) and complex post-traumatic stress disorder (CPTSD) (McLean & Gallop, 2003) A recent systematic review and meta-analysis by Boumpa et al (2022) set out to explore this association of CSA and lifetime diagnosis of psychiatric conditions (Boumpa et al., 2022).

### Aim of commentary

This commentary aims to critically appraise the methods used within the review Boumpa et al (2022) and expand upon the findings in the context of social work practice.

### Critical appraisal and methods of Boumpa et al (2022)

By applying an adapted version of the Joanna Briggs Institute (JBI) tool for critically evaluating systematic reviews, this systematic review achieved nine out of nine criteria (see table 1 for full critical appraisal). The primary concern was the interchangeable use of the description of the population of interest. While the abstract specifies the inclusion of only children and adolescents in the review, the methods section suggests a broader scope, encompassing children, adolescents, and young people. Furthermore, the introductory paragraph of the discussion implies that the findings relate to adolescence and young adulthood. It’s essential to clarify that the studies included in the review encompassed participants ranging from two-year-olds to 20-year-olds. Despite this the review was deemed to adequately synthesise the evidence in context to the question of interest. But it is important when interpreting these findings to be aware of this factor when assessing the external validity of these findings.

### Main findings of Boumpa et al (2022)

After duplicate removal 5,476 papers were identified for screening of which 30 papers were identified to be included in this systematic review (28 papers used within meta-analysis: total cohort of 28,693). The majority of studies only achieved 0 to 4 of the criteria of the Newcastle Ottawa scale with only two screened study scoring five out of a possible seven criteria. The most common issue was that no study demonstrated that the outcome of interest was not present at start of study and the studies were deemed not to be representative of the target population.

When meta-analysed using a random effects model there was a statistically significant increase odds of PTSD for girls (Odds Ratio [OR] = 2.38, 95% Confidence interval [Cl] 1.76 to 3.23) and boys (OR = 2.86, 95% CI 2.09 to 3.91) with a history of Child sexual abuse (CSA). For both comparisons, there was a negligible level of insignificant heterogeneity. In a meta-regression analysis, the moderating factor of gender was evaluated and found to have no impact on altering the odds of PTSD.

For those studies which did not differentiate between genders when combined there was a statistically significant increase odds of PTSD for individuals with a history of CSA (OR = 3.60, 95% CI 2.75 to 4.73). Upon evaluation for publication bias using the Egger’s test, no evidence of publication bias was detected. However, there was substantial statistically significant heterogeneity between studies (I^2^=63.2).

When compared the geographical regions of Canada/USA, Africa and Europe were not statistically significantly different. Despite there being no significant difference there was a notable increase in the odds of PTSD in people from Canada/USA (OR = 4.20, 95% CI 2.80 to 6.31). Although there was still substantial heterogeneity (I^2^ = 62.6%). Furthermore, the two studies which were meta-analysed from Europe demonstrated no statistically significant difference.

### Commentary

Using modified Grading of Recommendations, Assessment, Development, and Evaluations (GRADE) principles to evaluate the certainty in the estimates presented within this review the certainty of the estimates presented range from low to very low certainty (Borges Migliavaca, Stein, Colpani, Barker, Munn, & Falavigna, 2020). Concerning the estimates provided for studies that did not distinguish between genders, there was a low certainty of evidence suggesting that children, adolescents and young adults who have experienced CSA are 3 ½ times more likely to have PTSD compared to those who have not experienced CSA. This reduction in certainty is due to the low methodological quality of included studies, substantial heterogeneity, and reduce directness of the exposed group within the included studies. Despite this reduced certainty it is likely that children, adolescents and young adults who have experienced CSA are more likely to present with PTSD rather than their counterparts. Although the specific estimate of the increase in relative risk given in the review may not be accurate.

The effects of PTSD in young people can be substantial if not treated (Lewis et al., 2019). It can result in the individual being at higher risk to develop further complex psychiatric presentations and significant impairment (Lewis et al., 2019). It has been proposed that improve screening (Lewis et al., 2019) and training of professionals may facilitate in providing better and more responsive services for young adults with PTSD (Rosen, Ruzek, & Karlin, 2017). Therefore, it is essential that social workers serving this demographic to maintain up-to-date knowledge and training on PTSD screening and treatment. A recent survey of UK social workers revealed that while clinicians were generally confident in identifying in young people PTSD, they expressed less confidence when it came to providing treatment and support for individuals with the disorder (Finch, Ford, Lombardo, & Meiser-Stedman, 2020). As highlighted in the survey, the importance of training levels and adequate supervision emerged as significant factors that could enhance confidence levels in both the assessment and treatment of this condition (Finch et al., 2020).

Similarly, a previous valuation of professionals working with individuals with serious mental illness within England identified that they often lack confidence to talk about sexual harm (Hughes, Edmondson, Onyekwe, Quinn, & Nolan, 2018). The World Health Organization recommends that identifying abuse or maltreatment at an early stage leads to improved long-term outcomes for individuals (WHO, 2022). Furthermore, early identification and prevention of CSA could reduce the risk of PTSD from occurring (Lewis et al., 2019). Social workers are well placed to build trusting relationships with children, adolescents and young adults and create a safe core environment for CSA to be disclosed (Smith, 2017). On the rare occasion where CSA is disclosed it is often to a care professional who has a trusting and supporting relationship with the individual (Brooker, Hughes, & Lucock, 2019). Training specific to relationship based practice is fundamental to support earlier identification of, and prevention of CSA, which in turn had the potential to reduce the severity and impact of PTSD (Smith, 2017). National initiatives such as Research in Practice (RiP)(Research in Practice, 2024), NSPCC (NSPCC, 2024) and Banardos (Barnardos, 2024) provide training and practice guidance, commissioned by some local authorities and NHS trusts to upskill social workers in identifying CSA and steps to prevent it.

Models of PTSD treatments and therapies, such as the Trauma Focussed Cognitive Behavioural Therapy (de Arellano et al., 2014) or Eye Movement Desensitisation Reprocessing (EMDR) (Shapiro (Shapiro, 2014) report positive outcomes in the treatment of PTSD and should be considered when assessing training options. Likewise, training and knowledge exchange regarding locally available services that offer specialized interventions for individuals experiencing long-term mental health issues stemming from CSA are beneficial for social work. Organizations such as Trust House Lancashire (Trust House Lancashire, 2024), and the Magdalene Project in East Lancashire (The the Magdalene Project, 2024), which provide professional training and therapy for CSA victims, offer valuable support in this regard.

The results of the systematic review indicate that in studies where gender was specified, there was no significant difference observed in the likelihood of children, adolescents, and young adults who experienced CSA having PTSD between males and females. This conclusion was further supported by a Meta-Regression analysis, which found that gender, as a potential moderating factor, did not appear to correlate with an increased likelihood of PTSD. From a practice perspective this means that when screening for PTSD there shouldn’t be any perceived difference between males and females when considering the odds of PTSD occurrence within children, adolescents, and young adults who has experienced CSA. There was also no evidence that geographical location was an important moderating factor regarding odds of PTSD in children, adolescents, and young adults who experienced CSA. However, there was notable increase in odds within the United States compared to other geographical regions.

As noted in the review, a significant portion of the studies analyzed suffered from notable methodological shortcomings, particularly concerning the identification of the primary outcome. It is imperative that future research in this domain adopts a robust and comprehensive framework for identifying both instances of child sexual abuse and diagnoses of PTSD. Regrettably, the primary meta-analysis did not delve deeper into the substantial heterogeneity observed, merely examining geographical location and gender. Given this study variance, future investigations should explore other potentially influential moderating factors that could hold clinical significance. Moreover, the scarcity of studies conducted in middle to low-income countries underscores the necessity for research in these regions to ascertain whether this association holds true on a global scale.

## Figures and Tables

**Table 1:** Critical appraisal using the JBI critical appraisal checklist for systematic reviews (Boumpa et al 2022) and research syntheses (Aromataris, Fernandez, Godfrey, Holly, Khalil, & Tungpunkom, 2015).

JBI critical appraisal checklist items	Responses
1. Is the review question clearly and explicitly stated?	Yes, the question is clearly stated, and all key variables identified within the research question.
2. Were the inclusion criteria appropriate for the review question?	Yes - All criteria were relevant to the question of interest. However, it is important to note that there were some discrepancies regarding the aims of the review for what is stated in the abstract in context to the paper with the subgroup of young adults not being identified as a target population in the abstract but only in the main paper. Included studies were those who specifically reference childhood sexual abuse (CSA) and the subjects were aged 21 years or younger. PTSD had to be included as a positive or negative binary but could be via any diagnostic means. Studies had to be written in English, but were not limited by place, all countries were included. Three authors conducted Abstract and title screening independently.
3. Was the search strategy appropriate?	Yes - A thorough search strategy was deployed.
4. Were the sources and resources used to search for studies adequate?	Yes - 4 large relevant databases were used. Four databases were searched (PUBMED, EMBASE, PSYCINFO and GOOGLE SCHOLAR) from date of inception to 31st August, 2020. Additionally, all included studies citation lists were screened for relevant papers
5. Were the criteria for appraising studies appropriate?	Yes - A known method of appraisal was used, the Newcastle–Ottawa Scale (NOS), to assess the quality of the studies.
6. Was critical appraisal conducted by two or more reviewers independently?	Yes - Assessment of quality was undertaken by three reviewers in groups of two independently with verification by a third review.
7. Were there Methods to minimize errors in data extraction?	YES – Data extraction was undertaken by three reviewers in groups of two independently with verification by a third review.
8. Were the methods used to combine studies appropriate?	Yes – The study utilised random effects models to calculate pooled odds ratios with a confidence interval of 95%. Studies were analysed separated by each gender, and those containing both genders. A further subgroup analysis was performed of geographical location. Between study heterogeneity was assessed using the I^2^ statistic. Meta-regression analysis was performed to examine if the number of boys in the study affected the results.
9. Was the Likelihood of Publication bias assessed?	Yes - Egger’s statistical test was performed to assess possible publication bias.

## Key Points

- Low certainty evidence suggests that children, adolescents and young adults who have experienced CSA are 3 ½ times more likely to have PTSD compared to those who have not experienced CSA.
- There is no evidence of difference between males and females regarding odds of likelihood of children, adolescents and young adults who have experienced CSA having PTSD.
- Despite geographical location there is a strong association between CSA and PTSD in children, adolescents and young adults.
